# Seroprevalence and risk factors of *Chlamydia abortus* infection in free-ranging white yaks in China

**DOI:** 10.1186/s12917-015-0323-y

**Published:** 2015-01-20

**Authors:** Si-Yuan Qin, Si-Yang Huang, Ming-Yang Yin, Qi-Dong Tan, Guang-Xue Liu, Dong-Hui Zhou, Xing-Quan Zhu, Ji-Zhang Zhou, Ai-Dong Qian

**Affiliations:** College of Animal Science and Technology, Jilin Agricultural University, Changchun, Jilin Province 130118 PR China; State Key Laboratory of Veterinary Etiological Biology, Lanzhou Veterinary Research Institute, Chinese Academy of Agricultural Sciences, Lanzhou, Gansu Province 730046 PR China; College of Veterinary Medicine, Hunan Agricultural University, Changsha, Hunan Province 410128 PR China; College of Animal Science and Technology, Anhui Agricultural University, Hefei, Anhui Province 230000 PR China

**Keywords:** *Chlamydia abortus*, White yaks, Seroprevalence, Tibetans, China

## Abstract

**Background:**

*Chlamydia* is gram-negative obligate bacteria which causes a wide variety of diseases in humans and animals. To date, there are a few reports about the seroprevalence of *Chlamydia* and the risk factors associated with *Chlamydia* infection in yaks in the world. In this study, 974 blood samples were collected from white yaks (*Bos grunniens*) in Tianzhu Tibetan Autonomous County, Gansu province, northwest China from June 2013 to April 2014.

**Results:**

Antibodies against *Chlamydia abortus* were examined by the indirect hemagglutination (IHA) test, and 158 of 974 (16.22%) white yaks were seropositive for *C. abortus* antibodies at the cut-off of 1:16. The risk factors associated with seroprevalence were evaluated by a multivariate logistic regression analysis. Region, gender and age of white yak were left out of the final model, due to its insignificance in the logistic regression analysis (*P* > 0.05). However, season was considered as a major risk factor associated with *C. abortus* infection in white yaks.

**Conclusions:**

To our knowledge, this is the first survey of *C. abortus* seroprevalence in white yaks in China, which extends the host range for *C. abortus* and has important implications for public health and the local Tibetan economy.

## Background

*Chlamydia* spp. are obligate intracellular bacteria which are the etiological agents of chlamydiosis and cause a broad spectrum of diseases in animals and humans [1,2]. *Chlamydia* are among the main pathogens for infection of the eye and the urogenital and respiratory tracts in humans, and chronic and repeated infection may result in severe irreversible damage such as blindness (trachoma) and tubal infertility [1].

*Chlamydiaceae* has a single genus *Chlamydia* that currently comprises twelve species [3-6]. Previously studies indicated that some of them can infect cattle, including *Chlamydia pecorum*, *C. abortus*, *C. psittaci*, and *C. suis* [7]. *C. abortus* is one of the most important pathogens which lead to reproductive failure in yaks, especially in arctic-alpine areas in China and other countries. Furthermore, pregnant women can be infected through contacting with animals infected with *C. abortus*, so it is also a zoonotic pathogen [2]. Recently, *C. abortus* infection in cattle has been reported in many countries such as UK, Sweden, Germany, Jordan, Ireland, Switzerland, Slovak Republic and Poland [8-15], as well as China [16,17].

White yak is a semi-natural and rare breed of cattle in the world which only lives in Tianzhu Tibetan Autonomous County, Gansu province, northwest China with a condition of alpine hypoxia and low temperature [18]. Milk and meat of white yaks are the popular delicacy for local Tibetan people and other residents in Gansu Province. However, limited data about *C. abortus* infection was available in yaks all over the world [16,19], and no information on the prevalence of *C. abortus* in white yaks is available. The objective of the present survey was to investigate the seroprevalence and risk factors including region, gender, age and season of *C. abortus* infection in white yaks in Tianzhu Tibetan Autonomous County, northwestern China. The results may provide essential information to prevent and control *C. abortus* infection in white yaks.

## Methods

### Ethics statement

This study was approved by the Animal Ethics Committee of Lanzhou Veterinary Research Institute, Chinese Academy of Agricultural Sciences (Approval No. LVRIAEC2013-010). The white yaks from which the serum samples were collected were handled in accordance with good animal practices required by the Animal Ethics Procedures and Guidelines of the People’s Republic of China.

### The investigation site

The present study was carried out in Xidatan village and Zhuaxixiulong village in Tianzhu Tibetan Autonomous County. The county has an area of 7,149 square kilometers, and the vast majority of its land is more than 3,000 meters above sea level. The average temperature of this region is −8°C to 4°C. In this mountainous area, the free-range white yaks feed on local weeds and brook, which only live in Tianzhu Tibetan Autonomous County in China.

### Serum samples

A total of 974 blood samples were collected from white yaks in Tianzhu Tibetan Autonomous County of Gansu province between June 2013 and April 2014. The white yaks were randomly selected, blood samples of which were transported to the laboratory, kept at room temperature for 2 h and centrifuged at 3000 g for 10 min to separate clear serum. The serum samples were stored at −20°C until further analysis.

### Serological examination

A commercially available Indirect Hemagglutination Assay (IHA) kit (Lanzhou Veterinary Research Institute, Chinese Academy of Agricultural Sciences, Lanzhou, China) was purchased to test antibodies to *C. abortus* and it was carried out according to the manufacturer’s instructions as described previously [16]. The Ministry of Agriculture of the People’s Republic of China (NY/T 562–2002) has confirmed the sensitivity and specificity values for the testing kit used in this study. In brief, serum samples were added to 96-well V-bottomed polystyrene plates, which were diluted 4-fold serially beginning with 1:4 to 1:1,024. Each test was performed with positive, negative and blank controls, and serum samples which had positive reaction at dilutions of 1:16 or higher dilutions were considered positive for *C. abortus* antibodies.

### Risk factors

Information about age, gender, sampling season and geographical origin of yaks were collected from the owners of white yaks. The ages of sampled yaks were divided into four categories: yr ≤ 1, 1 < yr ≤ 2, 2 < yr ≤ 3 and yr > 3. The sampling seasons were distributed in spring (March to May), summer (June to August), autumn (September to November) and winter (December to February). Two regions and two genders of yaks were also analyzed in this study.

### Statistical analyses

Variables associated with *C. abortus* infection among white yaks of different geographical origins, gender, seasons and age groups were analyzed in a multivariable logistic regression model, probability (*P*) value < 0.05 was considered as statistically significant between factors and prevalence. Odds-ratios (OR) with 95% confidence intervals based on likelihood ratio statistics are reported. All statistical analyses were performed using the PASW Statistics 18.0 (SPSS Inc., IBM Corporation, Somers, NY).

## Results

In this study, serum samples were collected from a total of 974 white yaks (73 from Zhuaxixiulong village and 901 from Xidatan village) in Tianzhu Tibetan Autonomous County, Gansu province, northwest China. 158 out of 974 serum samples (16.22%) were seropositive for *C. abortus* by IHA test at the cutoff dilution of 1:16 (Table 1). The seroprevalence was 28.77% (21/73) in Zhuaxixiulong village and 15.21% (137/901) in Xidatan village. Seroprevalence of *C. abortus* infection in male and female white yaks were 17.30% (50/289) and 15.78% (108/685), respectively (Table 1). The ages of the examined white yaks varied from 0 year to 3 years or greater and the seroprevalence ranged from 12.17% to 20.57%. The seroprevalence in spring, summer, autumn and winter were 10.91%, 24.91%, 11.26% and 16.00%, respectively (Table 1). The antibodies titers were diverse in different regions, genders, ages and seasons with the most frequent level of 1:16 in 93 samples (58.86%), followed by 1:32 in 43 samples (27.22%), 1:64 in 16 samples (10.13%), 1:128 in 4 samples (2.53%) and 1:256 in 2 samples (1.27%) (Table 2).

**Table 1:** **Seroprevalence of** ***Chlamydia abortus*** **infection in white yaks associated with different factors in Tianzhu Tibetan Autonomous County, northwest China by indirect hemagglutination assay (IHA)**

**Biometric data**	**Category**	**No. tested**	**No. positive**	**Prevalence (%)**
Region	Zhuaxixiulong	73	21	28.77
	Xidatan	901	137	15.21
Sex	Male	289	50	17.30
	Female	685	108	15.78
Season	Spring	220	24	10.91
	Summer	273	68	24.91
	Autumn	231	26	11.26
	Winter	250	40	16.00
Age (yr)	0 < yr ≤1	115	14	12.17
	1 < yr ≤ 2	180	27	15.00
	2 < yr ≤ 3	175	36	20.57
	> 3	504	81	16.07
Total		974	158	16.22

**Table 2:** **Antibody titers of** ***Chlamydia abortus*** **infection in white yaks in Tianzhu Tibetan Autonomous County, China determined by indirect haemagglutination (IHA) test**

**Biometric data**	**Category**	**Antibody titers**					**No. positive**	**No. tested**	**Prevalence (%)**
		**1:16**	**1:32**	**1:64**	**1:128**	**1:256**			
Region	Zhuaxixiulong	10	7	2	1	1	21	73	28.77
	Xidatan	83	36	14	3	1	137	901	15.21
Sex	Male	24	18	7	1	0	50	289	17.30
	Female	69	25	9	3	2	108	685	15.78
Age (years)	≤ 1	11	1	2	0	0	14	115	12.17
	1 < yr ≤ 2	22	2	3	0	0	27	180	15.00
	2 < yr ≤ 3	15	15	5	1	0	36	175	20.57
	yr > 3	45	25	6	3	2	81	504	16.07
Season	Spring	18	3	3	0	0	24	220	10.91
	Autumn	17	8	1	0	0	26	231	11.26
	Winter	27	6	6	0	1	40	250	16.00
	Summer	31	26	6	4	1	68	273	24.91
	Total	93	43	16	4	2	158	974	16.22

According to forward stepwise logistic regression, region, age and gender of white yak were not significant in the logistic regression analysis (*P* > 0.05) and left out of the final model (Hosmer and Lemeshow goodness of fit test *P* = 1.00). Only one factor, season of collecting samples was considered as main risk factor to influence the seroprevalence significantly (Table 3).

**Table 3:** **Odds ratios for seasons of white yak are taken as risk factors for** ***Chlamydia abortus*** **seroprevalence in white yaks**

**Factor**	**Category**	**No. tested**	**No. positive**	**Prevalence (%)**	**OR (95% CI)**	***P*** **value**
Season	Spring	220	24	10.91	Reference	
	Autumn	231	26	11.26	1.04 (0.58-1.87)	0.907
	Winter	250	40	16.00	1.56 (0.90-2.68)	0.110
	Summer	273	68	24.91	2.71 (1.64-4.49)	<0.001

## Discussion

The epidemiological information regarding the prevalence of *C. abortus* in yaks are limited in the world, including China. There were described cases in yaks in India and China but no information on the prevalence of *C. abortus* in white yaks. The present study, therefore, aimed at estimating *C. abortus* seroprevalence in white yaks. The results revealed the presence of *C. abortus* in 16.22% of white yaks in the present study, which was lower than the value of 35% in yaks in India [19] and similar to the 17.66% seroprevalence in black yaks in Qinghai province, China [16]. The differences in the seroprevalence of *C. abortus* exposure in yaks in different regions could be related to differences in ecological and geographical factors including temperature, rainfall or landscape differences. In addition, sanitation and husbandry practices in yak production could be other reasons for the variation of prevalence.

The significant different seroprevalence observed may also be caused by the different methods, including antigen ELISA, IHA, conventional or multiplex PCR. However, IHA is considered as a simple, safe, and reliable means of testing *C. abortus* antibodies, which has been employed in previous serological investigations [16,20]. The sensitivity and specificity of the testing IHA kit have been validated by the Ministry of Agriculture of the People’s Republic of China, which demonstrates that the IHA is not only more efficient than the CFT but is also more inexpensive than the ELISA [21]. Therefore, it may be the most appropriate commercially available kit for detecting *C. abortus* infection in white yaks.

In the present study, statistical analysis showed that there was no significant difference in seroprevalence between male and female yaks (*P* > 0.05), suggesting that gender may not be a crucial factor for *C. abortus* infection in white yaks. The result is consistent with the studies of Chen et al. [16] in which they reported no association between sex and *C. abortus* prevalence in black yaks in Qinghai province. The highest prevalence (20.57%) was found in the 2 < years ≤ 3 age group, while the lowest prevalence was found in younger white yaks (12.17% in 0 < years ≤ 1 age group). Age of white yaks as a continuous variable was analyzed in the logistic regression model, the results showed that there is no significant difference in *C. abortus* seroprevalence in white yaks of different ages (*P* > 0.05), which is in agreement with a previous studies [16]. In addition, the geographical origin of white yaks is also not a risk factor associated with *C. abortus* seroprevalence through logistic regression analysis (*P* > 0.05), although the seroprevalence in Zhuaxixiulong village was much higher than that in Xidatan village. The number of yak samples collected from Zhuaxixiulong is much smaller than that in Xidatan, on account of small white yak population in Zhuaxixiulong village, which may have a significant effect upon the statistical tests used.

This study revealed that season is a significant risk factor associated with *C. abortus* prevalence due to different climates in different seasons, including diverse temperature, precipitation and humidity. The highest seroprevalence was in summer followed by winter, autumn and spring, and the higher seroprevalence in summer may be related to producing antibody following abortion. Abortion usually occurred in spring resulting in generating much antibody for resistance to *C. abortus* and the antibody titers would reach its highest peak in summer. After abortion, the rubbish and excretion of abortion may be contacted by other healthy white yaks, which increased the number of infected white yak in summer. Therefore, it is necessary to take effective measures to prevent the spread of *C. abortus* during reproductive seasons.

Logistic regression analysis showed that white yaks in summer (24.91%) had a 2.7 times higher risk of being seropositive compared to white yaks in spring (10.91%) (OR = 2.71, 95% CI = 1.64–4.49, *P* < 0.001), although no seasonal differences were found among other two seasons compared to spring (*P* > 0.05). Such significant season differences in prevalence may be associated with changes in climate conditions. The warm temperature and appropriate precipitation in summer are predicted to elevate the survival of *Chlamydia*, leading to increased transmission to white yaks in this area.

IHA is considered as a simple, fast and inexpensive method in the serological survey of *Chlamydia* infection which has been used to detect some kinds of antibodies against *Chlamydia* in several animal species [20,22-24]. 974 white yak samples were collected in Tianzhu Tibetan Autonomous County,where there are only approximately 49,400 white yaks [25]. Although survey results may not reflect the status of *C. abortus* infection in all the white yaks, our results could provide useful information for the effective prevention and control of chlamydiosis in white yaks.

## Conclusions

Results of the epidemiological study revealed a high *C. abortus* seroprevalence in white yaks from Tianzhu Tibetan Autonomous County, which can cause economic losses to the local cattle industry, and also pose a potential threat to local Tibetans health in this area. This report extends the host range for *C. abortus*, and provides basal information to prevent and control *C. abortus* infection in white yaks.

## Abbreviations

IHA: Indirect hemagglutination
*C. abortus*: *Chlamydia abortus*
*C. pecorum*: *Chlamydia pecorum*
*C. psittaci*: *Chlamydia psittaci*
*C. suis*: *Chlamydia suis*
OR: Odds-ratios
*P*: Probability
ELISA: Enzyme-linked immunosorbent assay
PCR: Polymerase chain reaction
CFT: Complement fixation test
CI: Confidence interval
